# Organoids as a Systems Platform for SCLC Brain Metastasis

**DOI:** 10.3389/fonc.2022.881989

**Published:** 2022-04-28

**Authors:** Vito Quaranta, Amanda Linkous

**Affiliations:** Department of Biochemistry, Vanderbilt University, Nashville, TN, United States

**Keywords:** small cell lung cancer, brain metastasis, systems biology, cerebral organoids, tumor microenvironment, tumor heterogeneity

## Abstract

Small Cell Lung Cancer (SCLC) is a highly aggressive, neuroendocrine tumor. Traditional reductionist approaches have proven ineffective to ameliorate the uniformly dismal outcomes for SCLC – survival at 5 years remains less than 5%. A major obstacle to improving treatment is that SCLC tumor cells disseminate early, with a strong propensity for metastasizing to the brain. Accumulating evidence indicates that, contrary to previous textbook knowledge, virtually every SCLC tumor is comprised of multiple subtypes. Important questions persist regarding the role that this intra-tumor subtype heterogeneity may play in supporting the invasive properties of SCLC. A recurrent hypothesis in the field is that subtype interactions and/or transition dynamics are major determinants of SCLC metastatic seeding and progression. Here, we review the advantages of cerebral organoids as an experimentally accessible platform for SCLC brain metastasis, amenable to genetic manipulations, drug perturbations, and assessment of subtype interactions when coupled, e.g., to temporal longitudinal monitoring by high-content imaging or high-throughput omics data generation. We then consider systems approaches that can produce mathematical and computational models useful to generalize lessons learned from *ex vivo* organoid cultures, and integrate them with *in vivo* observations. In summary, systems approaches combined with *ex vivo* SCLC cultures in brain organoids may effectively capture both tumor-tumor and host-tumor interactions that underlie general principles of brain metastasis.

## Introduction

Small cell lung cancer (SCLC) accounts for approximately 15% of all lung cancer cases. It is clinically more aggressive than non-small cell lung cancer (NSCLC), with over two-thirds of SCLC patients experiencing extensive-stage disease at the time of presentation (1). Extensive-stage disease can include liver and bone, and multiple lines of evidence indicate the brain as a frequent SCLC early metastatic site—an estimated 50-60% of SCLC patients will develop brain metastases during the course of their disease, an unfavorable event for which prophylactic cranial irradiation is often recommended (1–4). Rapid dissemination of SCLC tumor cells is at least partially responsible for the poor 5-year survival, less than 5%, with a median survival of only a few months (5–7). Improved understanding of these aggressive properties is an urgent need, which may lead to strategic approaches for preventing the formation of SCLC brain metastases.

There is significant evidence that the evolutionary trajectory of a tumor, including its metastatic properties, is shaped by the multidirectional interactions of tumor cells amongst themselves and with their microenvironment. In the case of SCLC, we and others have demonstrated that multiple SCLC subtypes and their intra-tumor interaction dynamics play a major role in treatment response and, ultimately, disease progression (8, 9). We hereby propose that investigations into a possible role for SCLC subtypes in metastasis are timely (8). To this end, both adequate experimental platforms and appropriate data analysis frameworks are in order. Recently organoids have become a popular, if technically onerous, *ex vivo* platform (10). Brain organoids can be derived from human embryonic stem (hES) cells, providing a consistent, genetically neutral platform for replicating metastatic invasion by cancer cells and evaluating their invasive and proliferative properties. In the case of SCLC, the availability of >100 well-characterized cell lines spanning every subtype, an abundance of patient-derived xenografts and circulating tumor cells invites studies to explore the breadth of metastasis biology in the context of organoid growth. Brain organoids can be propagated on a large scale, and therefore amenable to parallel testing of multiple desired perturbations of diverse, e.g., SCLC cell subtype combinations. An extremely desirable feature of organoid platforms is accessibility by imaging techniques, from bright-field to light-sheet microscopy and anything in between, in a time-resolved manner. Thus, the evolution of host-tumor cellular interactions can be recorded. Evolving interactions can also be resolved at the molecular level, e.g., with fluorescent live-cell reporters (11, 12) or multiplexed immunofluorescence (13, 14). The incipient advent of spatial single-cell transcriptomics and proteomics will undoubtedly enlarge the data bandwidth of organoids. This versatility is especially attractive for SCLC subtypes, which are variously characterized by genomic features, transcriptomic signatures, and proteomics. Inevitably, analytical challenges will arise to integrate such wealth of data at several biological scales. We further propose that systems approaches are uniquely suitable to meet these challenges. Systems biology aims to merge top-down with bottom-up data modeling approaches. For instance, from top-down models of cell population dynamics compelling hypotheses, e.g., subtype transitions, should be expected. Bottom-up approaches could produce mechanistic models of signaling networks that regulate cell-cell interactions, e.g., the NOTCH pathway, which tends to be mutated in SCLC.

In summary, this is an opportune juncture to invest in experimental platforms and analytical approaches that can capture both tumor-tumor and host-tumor interactions relevant to metastatic SCLC. Herein, we review the advantageous features of, and a systems perspective on, cerebral organoids as a model for SCLC brain metastasis.

## SCLC Tumor Subtype Heterogeneity

Inactivation of the tumor suppressors TP53 and RB1 is observed in over 99% of SCLC cases, together with extensive chromosomal rearrangements and a high mutational burden that identifies clusters as in other cancer types (15–19). Based on previous work by our laboratory and others, SCLC has been classified into five tumor cell subtypes characterized by eponymous transcription factors ASCL1 (SCLC-A and SCLC-A2), NEUROD1 (SCLC-N), YAP1 (SCLC-Y), or POU2F3 (SCLC-P) (8, 9, 20–23). The SCLC-A, -A2 and N subtypes exhibit transcriptomics and proteomics signatures consistent with neuroendocrine differentiation. In contrast, the SCLC-Y and -P subtypes are considered non-neuroendocrine (nonNE) by the same criteria (9). Distinct classification of individual tumors into one specific subtype is not straightforward, since multiple subtypes generally reside within a single tumor (24). In addition, subtype classification of single cells is a work-in-progress for the field, since many single cells in tumors or cell lines cannot be easily assigned to any of the subtypes. For instance, lack of expression of an eponymous transcription factor is frequently the case, preventing direct subtype assignment (18). Moreover, some single cells in culture exhibit morphology, and presumably transcriptomics, in-between NE (floaters) and nonNE (23), hindering clear-cut subtype designation. Recently, to obviate these issues, archetype analysis has been applied to SCLC transcriptomics data (18). Archetypes are idealized representations of a cell differentiation state in relation to a specific functional task (25). Groves et al. (18) showed that transcriptomics data from SCLC cell lines and tumors fit well in archetype space, and that current subtypes can be subsumed within this space. Depending on distance from archetype transcriptomics signatures, a single cell can be assigned a role as a specialist for an archetype task, or can be considered a generalist if about equidistant from one or more archetypes. The same nomenclature as subtypes is maintained, and generalist cells are presumed to be more adaptable since they exhibit task trade-offs between archetype tasks (18). This quantifiable definition of SCLC single-cell subtype (e.g., are they specialists, or generalists) is highly relevant to experimentation in organoids, since it is expected that SCLC cells may vary in their degree of plasticity and adaptation to changed microenvironmental conditions. For instance, they may respond to microenvironmental factors in a subtype-specific manner, and better adapt if they are generalist rather than specialist. Moreover, transitions between subtypes, or specialists and generalists, may further increase plasticity at the tumor population level.

A related issue in SCLC that may benefit from organoid platforms is the high level of circulating tumor cells (CTCc). In CTC-derived xenografts (26) from SCLC patients, treatment with cisplatin induced a global increase in intratumoral heterogeneity (26). Using platinum-sensitive and resistant CDX models, both baseline and single-cell RNAseq (scRNAseq) analyses were performed in CDXs and patient CTCs over the course of therapy (26). In addition to increased intratumoral heterogeneity, the onset of treatment resistance resulted in the emergence of distinct cellular populations defined by confirmed drug-resistance transcriptomics signatures (26). Such findings highlight a critical need to parse the relationship between platinum therapy response and SCLC transcriptional complexity in experimentally accessible platforms such as brain organoids.

Further adding to this complexity is the growing evidence that NE cancer cells can give rise to nonNE cancer cells that may promote tumor growth and enhance tumor survival during exposure to chemotherapeutic agents (27). For example, in genetically engineered mouse models (28) of SCLC, plastic NE cells can give rise to nonNE SCLC cells that exhibit vascular and mesenchymal features (27, 29, 30). These nonNE cells then use a paracrine signaling mechanism to enhance the metastatic potential of the neuroendocrine tumor cells (27, 29, 30). Organoid platforms are amenable to investigate these dynamics of subtype transitions with precise temporal definition.

Collectively, the complexity of SCLC intra-tumoral heterogeneity, dynamics of treatment response, and metastatic site adaptation warrant the adoption of *ex vivo* experimental organoid platforms that more closely represent the patient disease.

## Cerebral Organoids and SCLC Metastasis

Existing *in vitro* and *in vivo* models of tumor growth are often constrained in their capacity to emulate clinical disease. In the case of tumor metastasis, current model systems frequently lack a human tumor microenvironment and, thus, rarely exhibit the destructive colonization of the brain that is routinely observed in SCLC patients. Based on our previous work in primary brain tumor modeling (31–33), we created a realistic *ex vivo* model of the developing human brain that can be adopted to study brain metastasis. Through precise differentiation of human embryonic stem cells (hESCs), we can generate three-dimensional cerebral organoids that display stage-specific neural development and exhibit myelinated axons, dendrodendritic synapses, neurons and glial cells. In addition, these miniature brains demonstrate consistent choroid plexus formation and are positive for neural stem cell markers including Nestin, Musashi-1, and Sox2. The cerebral organoids also express high levels of Pax6—a vital transcription factor important for neural stem cell proliferation and neurogenesis. Furthermore, chronological studies of the cerebral organoids reveal reduced neurogenesis and heightened gliogenesis over time, with increased expression of glial fibrillary acidic protein (GFAP) as the organoids progressively mature in a process analogous to the aging mammalian brain (31).

**Figure 1:**
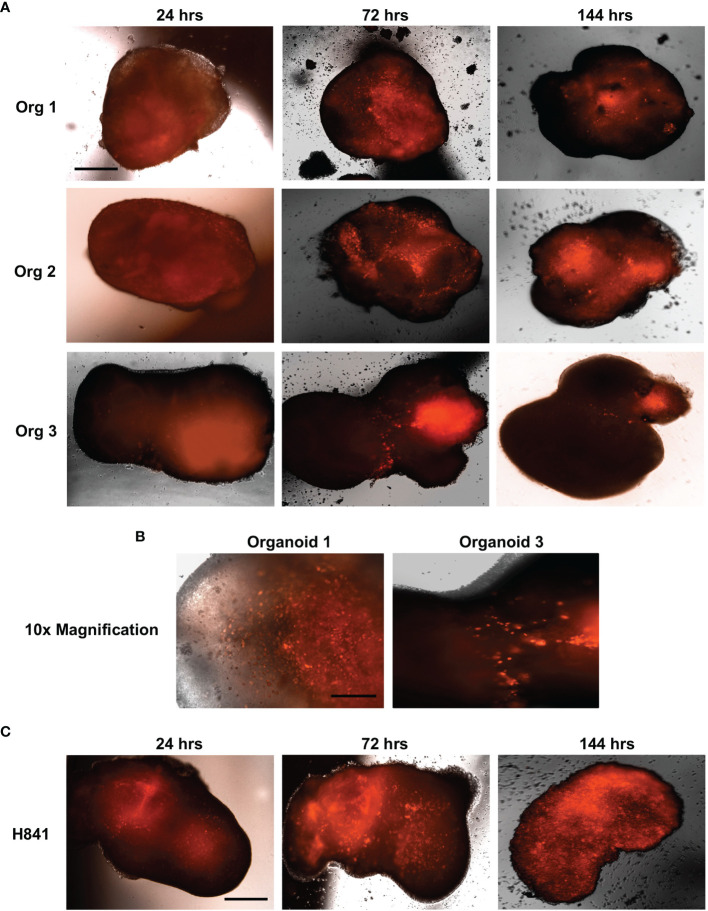
SCLC invasion of cerebral organoids. Shown are 4x immunofluorescent microscopic images of neuroendocrine H69 SCLC **(A)** or non-neuroendocrine H841 SCLC cells **(C)** labelled with mKate2 and co-cultured with cerebral organoids (100,000 cells per organoid); n = 3 organoids; scale bar, 650 μm. **(B)** 10x magnification of H69_mKate2 SCLC cell invasion of cerebral organoids at 72 hrs; scale bar, 275 μm. For all experiments, tumor cells were co-cultured with cerebral organoids for 24 hrs and imaged by fluorescent microscopy at 24, 72, and 144 hrs after co-culture.

Primary brain tumors grown within these cerebral organoids are highly representative of the patient disease and, remarkably, cerebral organoids preserve the cellular states and tumor plasticity found in the corresponding primary parental tumors (31, 33). Given the inherent heterogeneity and plasticity of SCLC (34–36), we believe cerebral organoids will also provide critical insight into SCLC metastatic brain tumor biology. Thus, we are now using our established tumor generation protocol to study SCLC invasion and colonization of the organoids and have demonstrated that both neuroendocrine (H69, SCLC-A subtype) and non-neuroendocrine (H841, SCLC-Y subtype) SCLC cell lines can successfully invade and form tumors within these miniature models of the human brain. **Figure 1** illustrates that SCLC cells labelled with the far-red fluorescent tag mKate2 invade the organoids within 24 hours of co-culture, and they continue to migrate and proliferate, resulting in diffuse infiltration of the entire organoid in less than a week. As there are many established differences between SCLC cells of a neuroendocrine subtype (-A, -A2, and N) vs. those with a non-neuroendocrine subtype (-Y, and -P), our future investigations will focus on the effects of the lung and brain organoid microenvironments on subtype composition for multiple neuroendocrine and non-neuroendocrine cell lines.

Cerebral organoids are also advantageous in both practicality and scalability. Tissue scarcity for primary and metastatic SCLC has been a long-standing obstacle to the molecular characterization of SCLC (37). Surgery is sometimes offered for very small SCLC with negative lymph nodes, but concomitant chemoradiation is often an alternative treatment plan (15). A paucity of tissue from patients with relapsed SCLC is even more apparent, as these patients often experience a rapid clinical deterioration (37). To circumvent the lack of available tumor tissue, efforts to unravel the genomic and transcriptomic landscapes of relapsed SCLC have recently focused on whole-exome and transcriptome profiling of SCLC patients through rapid research autopsy (37). Research autopsies were performed on five patients with metastatic SCLC. All had responded to first-line therapy with cisplatin or carboplatin and etoposide but relapsed within 1 to 5 months of completing the last treatment cycle (37). Each patient had multiple metastatic tumors from four to five different organs. Findings from the study revealed substantial clonal heterogeneity and found that specific clones were enriched in metastatic sites such as the brain and liver (37). Such results provide valuable insight into the clonal preference for specific metastatic niches. With limited tissue access as an ever-growing problem, however, especially during the current pandemic and accompanying restrictions for tissue acquisition, the SCLC field would benefit from a reliable and reproducible *ex vivo* platform for studying metastatic SCLC tumors in human tissue microenvironments. Cerebral organoid models are worth exploring to validate the feasibility of producing insightful data on SCLC adaptation mechanisms to a non-pulmonary microenvironment. It is to be emphasized that organoids are easily scalable to high-throughput assay conditions, i.e., from studying hundreds of metastatic brain *ex vivo* tumors from a wide variety of SCLC cell lines and PDXs, some general principle may emerge.

As mentioned above, the accessibility of organoids to experimental manipulations also enables longitudinal, time-resolved studies that are unwieldy in animals or essentially impossible in SCLC patients.

Lastly, organoid approaches to decode the mechanisms that govern organ-specific metastasis will not be limited to cancer cell autonomous properties (38). Tumor-derived molecular factors can also direct organotropism by preparing a pre-metastatic niche of specific organ sites. Extracellular vesicles (39) including exosomes (diameter <100 nm) contain proteins, RNA and/or DNA fragments and can facilitate pre-metastatic niche formation by mediating communication between tumor cells and the host component or by transferring their contents into recipient cells (40). Given the established importance of exosomes to cell migration and cancer metastasis (39, 41), the ability to isolate and identify exosomal proteins that are specific to normal, cerebral organoids and organoid tumors will serve as a powerful tool for exploring brain-specific metastasis from a mechanistic perspective. By comparing the exosomal profiles of normal organoids to those containing SCLC tumors, it should be possible to identify novel biomarkers for the early detection and subsequent treatment of SCLC brain metastases.

## Organoid-Enabled Systems Approaches

For more than 50 years, SCLC research has relied on traditional reductionist approaches to try and reverse the influences of oncogenic perturbations with pharmacological agents – yet, in spite of significant advances in our understanding of SCLC biology, the long-term prognosis for this disease remains dismal.

For example, despite the recent classification of SCLC into distinct subtypes, there is still much to be learned regarding phenotypic transitions from one cell subtype to another and how specific tumor microenvironments, such as the brain, impact subtype composition and stability over time. We submit that these transitions are best understood within a systems framework for SCLC tumors. Thus, at the systems level, the evolutionary dynamics of cancer cell populations can be evaluated, as they adapt to a metastatic niche. This is usually done by combining suitable datasets with appropriate mathematical models, in order to produce testable predictions and uncover fundamental principles governing the metastatic seeding process. It is reasonable to believe that local microenvironmental features play a key role in this process, as they interact with tumor cells. At the same time, tumor cells may produce their own factors that influence the formation of a niche and subsequent progression. The complexity of these interactions can grow fast, so that computational models become a requisite for obtaining quantitative information on the limiting steps in the process. However, to be rooted in reality, these models must incorporate experimental data, measurements, and observations in order to construct a relevant mathematical representation of the process of interest. Likewise, computer simulations of such models require an experimental platform to validate or falsify their predictions.

**Figure 2:**
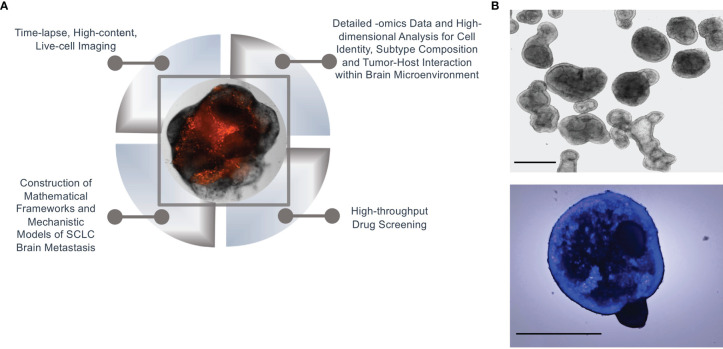
Systems biology approach in organoid SCLC tumors. **(A)** An illustration of a systems biology approach to SCLC brain metastasis using our cerebral organoid model. **(B)** Normal branching lung bud organoids (top) and SCLC invasion of branching lung bud organoids at 24 hours after co-culture (bottom); Hoechst-labelled H69 SCLC cells were co-cultured with cerebral organoids (100,000 cells per organoid); n = 3 organoids; scale bars, 650 μm.

In the case of SCLC metastasis, we propose that the normal brain microenvironment of cerebral organoids is a biologically meaningful model to satisfy both of these requisites. Thus, initial quantitative measurements and observations on SCLC invasion of brain organoids can set the stage to outline the physical process to be expressed in mathematical terms and construct a tentative model. Predictions from the model can then be verified on the same platform, by designing appropriate experiments. Specifically, cerebral organoids contain many cell types and structures found in the human brain (31, 33). Thus, they make it possible to interrogate the cell-cell interactions between SCLC tumor cells and the host component, as well as cellular interactions in-between the tumor cells themselves. The suitability for systems approaches of this platform is underscored by the multiple biologically and technically sophisticated angles that are possible for data generation, including: 1) Time-resolved, live-cell, high-content imaging to view the organoid tumors in their entirety; 2) Detailed -omics data of single tumor and normal host cells; 3) High-dimensional analysis of single-cell identity by Mass Cytometry; and, 4) High-throughput screening to evaluate the effects of therapeutic agents on tumor progression and subtype composition. Resulting datasets and biological findings can then be used to both construct and validate mathematical frameworks and mechanistic models of SCLC brain metastasis that model the dynamics of SCLC subtype composition, tumor progression, and response to treatment (**Figure 2A**). From a systems perspective, mathematical models rooted in organoid data may ultimately provide insights into adaptability at the single-cell level, robustness at the cell population level, and emergent properties of metastasizing SCLC cell ensembles.

Recently, the heterogeneity of SCLC subtypes has been placed in archetype space (18). The advantage of this analysis, performed on transcriptomics data, is manifold: i) rather than relying on expression of single transcription factors (TFs), often difficult to ascertain in single cells, SCLC archetypes are defined by robust transcriptomics signatures corresponding to known subtypes; ii) archetype signatures are evaluated for enrichment in functional tasks by GSEA (18) or similar methods, and these tasks can be matched to cancer hallmarks, or cell of origin functional phenotypes; iii) based on distance from archetypes, single cells can be assigned a specific task, and regarded as *specialists*; iv) single cells can also be regarded as *generalists* if they fall on a Pareto front that defines task trade-offs in-between archetypes.

Thus, SCLC cells may reside within a cell-state continuum rather than in discrete subtype clusters, and individual SCLC cells can increase their fitness and survival by trading off between defined tasks. SCLC cells can easily transition from specialists that optimize a single task to generalists that fall within the continuum, suggesting that these tumor cells become recalcitrant to treatment through a mechanism of phenotypic plasticity. In this dynamic cell-state continuum, intermediate generalists may represent either stem-like cells acting as a source for specialists at archetype vertices or cells in transition from one archetype vertex to another (18). Indeed, when calculating the Cell Transport Potential (CTrP), which is the expected distance of transition for each single cell, generalists across the continuum were likely to be high-plasticity transitioning cells. Specialists, however, were more likely to serve as end states with low plasticity – with the exception of SCLC-Y specialists which may act as a plastic source, if present (18). In a nutshell, generalist cells are suboptimal at multiple tasks and therefore presumably more plastic and adaptable to changing microenvironmental conditions. In contrast, specialists may fare poorly when challenged by perturbations. SCLC metastatic organoids, coupled with computational modeling, may be an ideal platform to investigate movement of SCLC cells in archetype space as they adapt to the brain microenvironment. It is hoped that fundamental insights may ensue, leading to new strategies to prevent or contain metastases.

It is also worth noting that the organoid platform can be adapted to studies at any biological scale. For example, we previously developed a Python-based algorithm, BooleaBayes, to infer mechanistic insights into the regulation of heterogenous SCLC subtypes at the level of transcription factor (TF) networks (9). Thus, BooleaBayes identified and ranked TFs that act as master regulators or destabilizers of individual SCLC subtypes, such as ISL1 (in NE cells) and TEAD4 (in nonNE cells) (9). BooleaBayes is essentially a machine-learning algorithm, and it will become increasingly relevant as -omics and cell identity data from SCLC tumors generated in cerebral organoids accumulate. Thus, BooleaBayes serves as a roadmap to select TFs to be genetically manipulated based on its own predictions (e.g., are they master regulators or destabilizers of a subtype)? and then experimentally tested in the organoids. Results will inform a new datapoint to further constrain the BooleaBayes model to hone in on the ground truth of subtype dynamics that support the metastatic process (9).

While operating at a holistic level, the ultimate goal of systems approaches is to reveal the vulnerabilities of a system. In the specific case of SCLC brain metastasis, this may come down to the activity of, e.g., a particular TF, tumor factor, or cell type. Any or all of these may be considered actionable targets. Due to their versatility in culture, brain organoids can also be a high-throughput screening platform of compounds for anti-metastatic effects. Traditional SCLC drug discovery strategies have largely focused on single targets, but resulting approved treatments have produced little improvements for SCLC patients (28). Given the plasticity of SCLC cells, addressing multiple targets at once, or sequentially, may be a better strategy that is suitably supported by a system-level understanding of tumor dynamics. However, discovering drug combinations effective even against only two targets becomes rapidly impractical due to combinatorial explosion, “the curse of dimensionality”. Systems-level knowledge can come to the rescue by identifying sensible nodes that could anchor large combinatorial screens. In this respect, the algorithm termed Multidimensional Synergy of Combinations (MuSyC) accurately quantifies drug synergy from high-throughput screening of drug combinations (42–44). MuSyC fulfills an unmet need in the field – as previous methods to measure drug synergy are often biased and fail to separate drug potency from drug efficacy. This is a real problem as there are diseases where potency is more important due to off-target toxicity and poor quality of life for the patient. In that particular scenario, the ability to select targets that improve drug potency could be the primary goal. For recalcitrant cancers like SCLC, however, efficacy of combination therapy is likely more desirable, stressing the need of a framework like MuSyC that can separate the two. MuSyC quantifies drug effect measured by any metric. Our preferred one, however, is the drug-induced proliferation (45) rate, which eliminates the temporal biases found in traditional, static point fractional killing assays (42–45). Using information from combinatorial screens, MuSyC quantifies synergy of potency separate from synergy of efficacy, ultimately revealing trends for entire drug classes which can be used to guide future screens and drug combination deployment (42–44). Aside from evaluating novel drugs, repurposing FDA-approved drugs is another possibility. For brain metastases, prophylactic or palliative cranial irradiation remains the only, often undesirable treatment due to brain function impairment. System-level knowledge gained from the organoid SCLC metastasis platform may open new avenues of MuSyC-based combination drug discovery specific to metastasis, due to the suitability of organoids to high-throughput drug screening.

There is a growing consensus that systems biology is a foundational precursor to precision medicine and will ultimately help predict which patients will benefit, or not, from a specific treatment. In particular, evolutionary dynamics of cell transitions and cell-cell interactions may be fruitful lines of investigation. One mystery of SCLC brain metastasis is whether tumors of a specific subtype are more likely to circulate, invade, and proliferate at distant sites compared to tumors of another subtype(s). Furthermore, once metastatic cells reach the brain, how are they able to survive in a microenvironment that is so intrinsically different from the lung? For instance, do the transcriptomic profiles and tumor subtypes change when SCLC cells are grown in a lung microenvironment vs. a brain microenvironment? Based on an existing protocol (46), we have recently developed a modified method to generate lung organoids to aid in our comparison of brain and lung microenvironments. Our initial results demonstrate that SCLC cells readily invade and proliferate in these organoids as well (**Figure 2B**). Evaluating these and other key questions at the single-cell and cell-population level will allow us to more accurately model the tumor plasticity and dynamics of cellular relationships that characterize this aggressive disease. It is our hope that this multi-tiered systems approach for understanding gene network development and perturbations will enable the discovery of breakthrough treatments for SCLC brain metastasis.

## Discussion

SCLC is a highly heterogeneous tumor defined by early spread, inevitable relapse, and extremely poor prognosis. There is a growing consensus within the field that heterogeneous SCLC tumor subtypes and their interaction dynamics form a robust SCLC tumor ecosystem adaptable to perturbations and treatment. The combined effort of many laboratories in the SCLC field has unraveled SCLC heterogeneity and classified SCLC into five subtypes: SCLC-A1, -A2, -N, -P, and -Y (9). However, no comprehensive data currently exists regarding the prevalence of subtypes in human SCLC brain metastases. Given that SCLC patients ultimately die from systemic disease, there is a considerable unmet need for a humanized model of SCLC brain metastasis.

Previous studies of tumor metastasis have often relied upon cell autonomous *in vitro* cell models that provide an incomplete representation of metastatic disease. Cancer cells are not self-reliant, however, but rather innately interdependent systems characterized by complex interactions with their host and one another (31). While transgenic mouse technology—as well as patient-derived xenografts—address some aspects of the host-tumor component and have provided great insight into the biology of metastatic growth, these *in vivo* models are fundamentally limited by shortened survival times and the inherent unknown interspecies differences between human cancer and murine host cells. Biological investigations of SCLC metastasis may, therefore, benefit greatly from the study of tumor growth within the human brain microenvironment of the organoids. Using a systems biology approach to interrogate single cell identity, dynamic cell-cell interactions, and potential biomarker identification, a cerebral organoid model may pinpoint subtype-specific vulnerabilities that we can exploit to thwart the systemic spread of this devastating tumor.

## Data Availability Statement

The original contributions presented in the study are included in the article/supplementary material. Further inquiries can be directed to the corresponding author.

## Author Contributions

AL designed and executed the experiments. VQ and AL contributed to the conception and drafting of the manuscript. All authors reviewed and approved the final version.

## Funding

VQ and AL received funding from the National Institutes of Health U54CA217450 and U01-CA215845.

## Conflict of Interest

The authors declare that the research was conducted in the absence of any commercial or financial relationships that could be construed as a potential conflict of interest.

## Publisher’s Note

All claims expressed in this article are solely those of the authors and do not necessarily represent those of their affiliated organizations, or those of the publisher, the editors and the reviewers. Any product that may be evaluated in this article, or claim that may be made by its manufacturer, is not guaranteed or endorsed by the publisher.
